# Insight Into Illness Among Inpatients in a Forensic Service - a Study From Dundrum Hospital as Part of the Dforest Study

**DOI:** 10.1192/bjo.2022.222

**Published:** 2022-06-20

**Authors:** Sean Murray, Mary Davoren, Harry Kennedy

**Affiliations:** Central Mental Hospital, Dundrum, Dublin, Ireland

## Abstract

**Aims:**

We endeavoured to ascertain if using a specific tool rating insight adds benefit over and above the insight ratings on violence risk assessment or recovery based tools currently in use and to see if they may be helpful in guiding clinical decision making.

**Methods:**

A cross sectional study of 104 forensic in-patients was completed. All current inpatients were rated for self-rated and clinician-rated insight using the VAGUS tool, a validated and reliable measure of insight into psychotic symptoms. All participants completed the self-rated scale independent of the clinician to avoid bias. Patients were also rated with the HCR-20, the Dundrum-3 and Dundrum-4, and the PANSS measures. Patients’ scores on the VAGUS tool and the other tools were compared to ascertain if any correlations could be identified.

**Results:**

Higher scores on the VAGUS tool were associated with a greater degree of insight into psychotic symptoms. Clinician and self-ratings of insight on the VAGUS tool were different from but complimentary to the ratings for insight on the HCR-20 (r = 0.480, p = <0.001), the DUNDRUM-3 (r = 0.491, p = <0.001) and DUNDRUM-4 (r = 0.265, p = 0.041). An inverse relationship between the VAGUS scores and the scores on the PANSS measures (r = 0.452, p = <0.001) was found, correlating lower levels of insight with a higher degree of positive and negative psychotic symptoms. There was also a correlation between greater insight and progress through the care pathway to lower secure wards.

**Conclusion:**

Using a specific tool to rate insight adds benefit over and above the insight ratings on other tools currently in use and may be helpful in guiding clinical decision making in the forensic setting.

